# Clinically Isolated Syndromes Suggestive of Multiple Sclerosis: An Optical Coherence Tomography Study

**DOI:** 10.1371/journal.pone.0033907

**Published:** 2012-03-20

**Authors:** Celia Oreja-Guevara, Susana Noval, Juan Alvarez-Linera, Laura Gabaldón, Beatriz Manzano, Beatriz Chamorro, Exuperio Diez-Tejedor

**Affiliations:** 1 Neuroimmunology and Multiple Sclerosis Unit, Department of Neurology, Idipaz Health Research Institute, University Hospital La Paz, Madrid, Spain; 2 Neuro-Ophthalmology Unit, Department of Ophthalmology, Idipaz Health Research Institute, University Hospital La Paz, Madrid, Spain; 3 Department of Radiology, Hospital Ruber Internacional, Madrid, Spain; 4 Department of Neurology, Hospital de Denia, Alicante, Spain; Institute Biomedical Research August Pi Sunyer (IDIBAPS) - Hospital Clinic of Barcelona, Spain

## Abstract

**Background:**

Optical coherence tomography (OCT) is a simple, high-resolution technique to quantify the thickness of retinal nerve fiber layer (RNFL), which provides an indirect measurement of axonal damage in multiple sclerosis (MS). This study aimed to evaluate RNFL thickness in patients at presentation with clinically isolated syndromes (CIS) suggestive of MS.

**Methodology:**

This was a cross-sectional study. Twenty-four patients with CIS suggestive of MS (8 optic neuritis [ON], 6 spinal cord syndromes, 5 brainstem symptoms and 5 with sensory and other syndromes) were prospectively studied. The main outcome evaluated was RNFL thickness at CIS onset. Secondary objectives were to study the relationship between RNFL thickness and MRI criteria for disease dissemination in space (DIS) as well as the presence of oligoclonal bands in the cerebrospinal fluid.

**Principal Findings:**

Thirteen patients had decreased RNFL thickness in at least one quadrant. Mean RNFL thickness was 101.67±10.72 µm in retrobulbar ON eyes and 96.93±10.54 in unaffected eyes. Three of the 6 patients with myelitis had at least one abnormal quadrant in one of the two eyes. Eight CIS patients fulfilled DIS MRI criteria. The presence of at least one quadrant of an optic nerve with a RNFL thickness at a P<5% cut-off value had a sensitivity of 75% and a specificity of 56% for predicting DIS MRI.

**Conclusions:**

The findings from this study show that axonal damage measured by OCT is present in any type of CIS; even in myelitis forms, not only in ON as seen up to now. OCT can detect axonal damage in very early stages of disease and seems to have high sensitivity and moderate specificity for predicting DIS MRI. Studies with prospective long-term follow-up would be needed to establish the prognostic value of baseline OCT findings.

## Introduction

A clinically isolated syndrome (CIS) involving the optic nerve, spinal cord, brainstem or other portions of the brain, is the most frequent initial presentation of multiple sclerosis (MS). Several studies have tried to identify the risk factors associated with the development of clinically definite MS (CDMS) after a first acute demyelinating attack [1]–[11]. The presence and extent of lesions on baseline magnetic resonance imaging (MRI) of the brain is strongly related to the probability of developing MS [1], [2], [5], [9], [10]. In addition, due to its high sensitivity in detecting disease-related abnormalities, MRI has been formally included in the diagnostic work-up of MS patients, to exclude alternative diagnoses and aid in the demonstration of disease dissemination in space (DIS) and time (DIT), which are the main criteria for a diagnosis of CDMS [12], [13].

Post-mortem [14], [15] and MRI [16]–[18] studies have demonstrated early axonal loss in MS patients. Since axonal damage is a key contributor to the clinical manifestations of the disease and to the development of clinical disability over time, biomarkers of early axonal degeneration in patients at presentation with CIS would therefore be of great interest.

Monitoring axonal loss has become a priority in MS. It has recently demonstrated that analysis of the retinal nerve fiber layer (RNFL) thickness may be useful to detect degenerative process in the central nervous system [19], [20]. Optical coherence tomography (OCT) has emerged as a simple accurate noninvasive technique that can be used to measure RNFL thickness. Several studies have consistently demonstrated that OCT is a useful tool to detect retinal axonal loss following an acute episode of optic neuritis (ON), a very common, often initial symptom of MS [21]–[25]. In addition, this technique can help to detect subclinical axonal loss, as suggested by the demonstration of a decreased RFNL thickness in fellow ON eyes or reduced RFNL thickness in MS patients without a history of ON [26]–[28].

The aim of this study was to measure RNFL thickness in patients at presentation with CIS suggestive of MS and to investigate the relationship between RNFL thickness abnormalities and clinical manifestations at disease onset, MRI features according to the International Panel (IP) criteria for DIS [14] and cerebrospinal fluid (CSF) profile.

## Methods

### Ethics statement

The study was approved by the local Ethics Committee (CEIC Hospital La Paz) and written informed consent was obtained from all subjects. The study complies with the guidelines of the Declaration of Helsinki.

### Patients

All consecutive patients with a single episode of CIS attending the Neurology Department of our center during 2008 were invited to participate in this study.

The diagnosis of ON was based on clinical criteria, including visual loss in the affected eye, relative afferent pupillary defect, a visual field defect and pain that increases with eye movement [29]. Exclusion criteria included the presence of an ocular pathology other than ON, intraocular pressure higher than 21 mmHg and a refractive error greater than 5.0 diopters (D) of spherical equivalent or 3.0 D of astigmatism in either eye. CIS with a clinical onset other than an ON were diagnosed on the basis of clinical criteria and, when needed, confirmed by the presence of lesions on brain or spinal cord MRI. Appropriate investigations were carried out as necessary to exclude alternative diagnoses, and all patients were carefully interviewed for a previous demyelinating event. Patients treated with corticosteroids for relapses before OCT and lumbar puncture examination were not included.

All patients underwent a complete neurological examination (within 7 days from symptoms onset) and neurological disability was measured using the Expanded Disability Status Scale (EDSS) [30]. Within the first two months from the attack, all the subjects underwent lumbar puncture (whenever possible) and brain and spinal cord MRI.

### MRI

The following MRI sequences of the brain were acquired using a 3 Tesla scanner (GE, USA): a) dual-echo axial variable echo fast spin echo (FSE): repetition time (TR) = 2940 ms; first echo time (TE) = 10 ms; second TE = 110 ms; echo train length (ETL) = 16, FOV = 24, 44 slices, slice thickness = 3.0 mm, interleaved slices, matrix = 256×256), b) axial 3D T1 Inversion recovery (IR) Prep: first TE = 10 ms; T1 = 750 ms; TR = 10 ms, flip angle = 12, FOV = 24, slice thickness = 1 mm, 24 slices, matrix = 240×240), c) axial fast fluid attenuated inversion recovery (FLAIR): TR = 9000, TE = 95, T1 = 2200; 3-mm-thick slices, FOV = 24, d) pre- and post-contrast T1-weighted conventional spin-echo (TR = 680 ms; TE = 14 ms, FOV = 24, slice thickness = 3.0, interleaved), 5 minutes after the intravenous administration of 0.1 mmol/kg gadopentetate dimeglumine.

The following sequences were also acquired for cervical cord: a) sagittal T2 FSE (TE = 99, TR = 3000 ms; ETL = 24; 4-mm-thick slices with an interslice gap of 0.4 mm), b) sagittal T1 FSE (TE = 22, TR = 575; ETL = 3; 4-mm-thick slices with an interslice gap of 0.4 mm), c) sagittal PD-weighted (TE = 38, TR = 2000; TE = 38; ETL = 8; 4-mm-thick slices with an interslice gap of 0.4 mm) and an oblique two-dimensional GE (TR = 300; FOV = 20, 4-mm-thick slices with an interslice gap of 1 mm) was a post-contrast T1w scan acquired for the cord.

Brain and cord MRI scans were assessed by two radiologists blinded to neurological and ophthalmological examinations and the fulfillment of IP criteria for DIS [31]. According to the original IP criteria, the presence of two brain MRI lesions consistent with MS together with positive oligoclonal bands (OCB) was also considered as DIS.

### Ophtalmologic evaluation

All study patients underwent a complete ophthalmologic evaluation, including best corrected Snellen visual acuity (VA), biomicroscopy of the anterior and posterior segments, funduscopy, intraocular pressure measurement and Humphrey visual field testing, using the Swedish Interactive Threshold Algorithm standard 24-2 strategy (Carl Zeiss Meditec, Dublin, CA). Affection of the visual field was defined according to the Optic Neuritis Trial criteria [32] and quantified using mean deviation (MD) (expressed in decibels [dB]).

OCT scanning was performed within the first month from the onset of the clinical episode with the Stratus OCT (Carl Zeiss Meditec, Dublin, CA) after induction of pharmacological mydriasis. Image acquisition was performed with the Fast Retinal Nerve Fiber Layer (RNFL) Thickness (3.46) and RNFL thickness values (measured in micrometers [µm]) were obtained using the RNFL Thickness Average Analysis protocol. The Stratus OCT built-in software compares a color-coded graph that displays the RNFL measurements with the age-matched data of a normalized database. Assuming a normal distribution of the RNFL, the thickest 5% of measurements are colored white (white >95%) and the thinnest 1% of measurements fall in the red area. Measurements in red are considered outside normal limits (red <1%, outside normal limits). Five percent of measurements fall in the yellow area or below (1%≤ yellow <5%, suspect) and 90% of measurements fall in the green area (5%≤ green ≤95%). Thickness values falling into the green area are considered normal. The yellow area marks thickness values that are 5% or less than all thickness values measured in the normative database. Thickness measurements in the red area are considered pathologic.

Sensitivity and specificity were calculated for RNFL thickness for the quadrant sectors at p<5% and p<1% cut-off values.

CIS patients were divided into two groups according to their clinical onset: CIS onset with ON (CIS-ON) and CIS onset without ON (CIS-nON). In CIS-ON patients, the eye which had suffered ON was defined as affected eye and was studied independently from the fellow eye. Left eyes of CIS-nON patients and fellow eyes of CIS-ON patients were grouped as unaffected eyes to include only one eye of each patient and therefore, avoiding design bias.

Statistical analysis was performed using the SPSS 12.0 program for Windows (SPSS Inc., Chicago, IL, USA). Visual acuity (VA) was measured in Snellen and expressed as a decimal value, but it was transformed to LogMAR system to be analyzed. VA was considered normal when it was of 1 expressed by decimal scale or 0 by LogMAR system. The Shapiro-Wilk test was used to verify if the distribution of variables values fit the normal distribution. Nonparametric tests were applied since most variables were not normally distributed. The Wilcoxon test was used to analyze differences between the eyes of each patient. A Mann-Whitney test was used for independent samples. The total level of significance was set at 0.05.

## Results

Twenty-four patients with CIS were recruited, including 8 (33%) patients with unilateral ON, 6 (25%) with spinal cord syndrome (myelitis), 5 (21%) with brainstem symptoms and 5 (21%) with sensory and other syndromes. There were thirteen (54%) women and 11 (46%) men, with a median age of 38 years (range 19–57 years). Their EDSS score ranged from 0.0 to 4.0, with a median of 1.0.

Lumbar puncture was performed in 21 patients because three of them refused. Oligoclonal IgG bands were found in 12 (57.1%) patients and the IgG index was increased in 10 (47.6%) patients.

Eight patients (33%) fulfilled IP criteria for DIS, whereas 8 patients had normal brain MRI scans. Cervical MRI was pathological in all 6 patients with spinal cord CIS and in two patients with DIS.

Nine of the patients who underwent CSF analysis (37.5%) showed two or more MRI lesions consistent with MS and positive oligoclonal bands.

Two CIS-ON patients had an anterior and 6 a retrobulbar form of ON, defined by the presence or absence of edema of the optic nerve head respectively. In the 8 patients with ON-affected eyes, mean VA was 0.36 (SD 0.31) and all presented affected visual fields, with a MD of −14.62 dB (SD 7.82). All the fellow eyes of CIS-ON patients had normal VA (p = 0.03) and visual fields were affected in 5 out of 8 (62.5%), with a MD of −2.22 dB (SD 2.31) (p = 0.01).

Visual acuity was normal in 21 out of 24 (87.5%) of unaffected eyes; it was 0.8 in two and 0.5 in one eye. Eight (33.3%) unaffected eyes of CIS-nON patients and ON patients had abnormal visual fields, with MD of −2.64 dB (SD 4.82).

Visual acuity was normal in 21 out of 24 (87.5%) of unaffected eyes; it was 0.8 in two and 0.5 in one eye. Five right eyes and 3 left eyes had abnormal visual fields, with a MD of −2.22 dB (SD 2.31) and −2.35 dB (SD 4.81), respectively.

RNFL thickness analysis provided the following results: 54.2% of all patients and 56.3% of the CIS-nON patients presented at least one quadrant of an optic nerve with a decreased RNFL thickness. Mean RNFL thickness was 142.13 µm (SD 13.56) in anterior ON eyes and it was 101.67 µm (SD 10.72) in retrobulbar ON eyes (p = 0.05). Mean RNFL thickness was 96.93 (SD 10.54) in unaffected eyes of CIS-nON patients and ON patients.

Table 1 shows mean RNFL thickness and the color assigned by the age-normalized Stratus-OCT database for each group of eyes.

**Table 1:** Mean RNFL thickness and its color assigned by the normalized Stratus-OCT database according to age and proportion of eyes with at least one quadrant P<5% and P<1% cut-off values.

	CIS-ON patients		CIS-nON patients	
	Affected eyes	Fellow eyes	Right eyes	Left eyes
	(N = 8)	(N = 8)	(N = 16)	(N = 16)
**Mean RFNL thickness ± SD (µm)**	101.67±10.72	97.44±13.53	92.58±9.49	96.67±9.21
**RFNL thickness color, n (%)**				
White	0 (0)	0 (0)	0 (0)	0 (0)
Green	6 (75)	7 (87.5)	13 (81.3)	15 (93.8)
Yellow	0 (0)	1 (12.5)	2 (12.5)	1 (6.3)
Red	0 (0)	0 (0)	1 (6.3)	0 (0)
**At least one quadrant at p<5% and p<1% cut-off values, n (%)**				
≥1 quadrant p<5%	3 (37.5)	3 (37.5)	9 (56,3)	3 (18.8)
≥1 quadrant P<5%	0	2 (25)	5 (31.3)	1 (6.3)

CIS: Clinically isolated syndrome; N: Number of eyes; ON: optic neuritis; RNFL: retinal nerve fiber layer.

Three of the 6 patients with myelitis had at least one abnormal quadrant in one of the two eyes. Three of the 8 (37.5%) ON affected eyes and 6 of 24 (25%) unaffected eyes presented at least one quadrant with a thickness at a p<5% cut-off value. Three of 24 (12.5%) unaffected eyes presented at least one quadrant with a thickness at a p<1% cut-off value. When we analyzed the results by patients, 13 (54.2%) and 7 (29.2%) patients had at least one quadrant with a thickness at a p<5% and a p<1% cut-off values, respectively.

Crosstabulation between OCT measures and MRI criteria and alternative criteria (at least two MRI lesions and OCB presence) for DIS are shown in Table 2. Eight CIS patients fulfilled DIS MRI criteria and 9 patients met alternative criteria for DIS based on MRI and OCB presence, Six out of 8 patients who fulfilled MRI criteria for DIS presented at least one quadrant with a RNFL thickness of less than 5% than all thickness values measured in the normative database. The presence of at least one quadrant of an optic nerve with a RNFL thickness at a p<5% cut-off value had a sensitivity of 75% and a specificity of 56% for predicting DIS according to the MRI Barkhof criteria. Specificity increased to 81% whereas sensitivity decreased to 50% when the cut-off value was set at p<1%. Sensitivity decreased to 67% at the p<5% cut-off value and to 33% at the p<1% cut-off value according to MIR criteria and OCB presence. Specificity was 58% at the p<5% cut-off value and increased to 67% at the p<1% cut-off value. Table 3 shows the sensibility and specificity for OCT findings according to 3/4 Barkhof criteria and alternative criteria based on MIR evidence and OCB presence.

**Table 2:** Crosstabulation between the two different criteria of spatial dissemination applied and OCT findings.

	Quadrants <5%		Quadrants <1%		
DIS MRI criteria	None	≥1	None	≥1	Total
Fulfilled	2	6	4	4	8
Not fulfilled	9	7	13	3	16
Total	11	13	17	7	24

Alternative criteria	Quadrants <5%		Quadrants <1%		
(OCB+ at least two lesions in MRI)	None	≥1	None	≥1	Total
Fulfilled	3	6	6	3	9
Not fulfilled	5	7	8	4	12
Total	8	13	14	7	21

DIS: dissemination in space; OCB: oligoclonal bands.

**Table 3:** Sensibility and specificity for OCT findings according to MIR Barkhof criteria and MIR and OCB criteria for DIS.

	¾ Barkhof MIR criteria		MIR and OCB criteria	
	<5%	<1%	<5%	<1%
**Sensitivity** (%)	75	50%	66.67%	33.33%
**Specificity** (%)	56.25%	81.25%	58.33%	66.67%

## Discussion

In patients diagnosed with MS it has been demonstrated that axonal loss occurs in the early stages of the disease. Therefore, a large effort has been focused on early detection of patients with high risk of developing MS at the first CIS in order to start early immunomodulatory treatment to reduce the accumulation of irreversible axonal loss.

OCT has demonstrated to be a useful tool to detect axonal loss as a thinning of the RNFL following an initial episode of ON. To our knowledge only one study have addressed the use of OCT on patients with other types of CIS different from ON, like myelitis or brainstem syndromes [21]. RNFL thickness increase in acute anterior ON and progressive axonal loss in anterior and retrobulbar ON has already been demonstrated [21], [33]. Mean RNFL thickness in the fellow non-affected eyes has ranged between 92.9 µm (SD 11.4) to 99.8 µm (SD 32.50) in studies focused on patients with isolated ON [23], [24], [34]. Statistical significant differences have not been found when compared non-affected eyes with control eyes, despite that their RNFL thickness mean values were above 100 µm. [25], [34]–[36]. Most studies have been unable to demonstrate a significant thinning of the RNFL of the fellow eyes when compared to healthy controls.

In our study, mean RNFL thickness was 101.67 µm and 97.44 µm in affected and fellow non affected eyes of the CIS-ON patients respectively. The increased thickness obtained in affected eyes could be explained by the presence of two anterior forms since RNFL thickness increases in optic nerve edema [37], [38]. Mean RNFL thickness was 92.58 and 97.44 µm in right CIS-nON eyes and fellow ON-eyes respectively, suggesting subclinical retinal axonal damage in CIS patients that occurs in the absence of optic ON. The findings are consistent with those of previous studies that described retinal axonal loss in patients with MS without previous ON history when compared with a healthy control population [27], [28], [39].

When we analyzed the color-code ordinal scale of RNFL thickness, it was found that only 4 out of 48 eyes had a mean RNFL thickness below the 5% cut-off value. This finding is consistent with previous absolute values in control groups [25], [34]–[36]. However, in our study, 18 out of 48 eyes had at least one quadrant in which RNFL thickness was below the 5% cut-off values and RNFL thickness was below the 1% cut-off values in at least one quadrant in 8 out of 48 eyes.These results suggest a mild axonal loss present in CIS even within the first week after its presentation. In brain studies, performed with proton magnetic resonance spectroscopy, diffusion tensor MRI and magnetization transfer, axonal damage has been demonstrated in patients presenting with CIS at baseline [17], [18], [40].

To our knowledge, this is the first study to demonstrate early retinal axonal layer thinning detected by OCT in patients who have suffered from any type of CIS, including patients with isolated myelitis. As expected, this thinning was mild but identifiable by the software of the Stratus-OCT.

At the moment, the presence and extent of demyelinating lesions in the MRI of patients with CIS is the main predictor of conversion to CDMS. Retinal axonal thinning present at the time of CIS presentation might be considered a signal of wider subclinical axonal damage and early neurodegeneration. Since the current study was conducted within the first two months of CIS presentation, we could only consider OCT and MRI pathological findings as measures of dissemination in space. Our results are in contrast to the only available study using OCT on CIS patients [41] that did not revealed retinal axonal loss at the earliest clinical stage of MS and did not predict conversion to MS at 6 months. Nevertheless, our study cannot be directly compared with the abovementioned study due to a substantial difference in disease duration. In this previous study, patients had a disease duration up to one year whereas our study population consisted of CIS patients who underwent OCT in the first month and MRI and lumbar puncture in the first two months. Additionally, the study population was very different to our CIS group since 23% of the patients had clinically definite MS and 71% presented dissemination in space according to the revised McDonald criteria. Nevertheless, it was shown that 25% of the patients had atrophy in at least one quadrant and 4 patients in two quadrants, which reflects a slight axonal damage.

Six out of 8 patients who fulfilled DIS MRI criteria presented at least one quadrant with less than 5% probability of normal RNFL thickness. This provides a sensitivity of 75%, which decreased to 50% when a higher probability of real RNFL atrophy is considered. However, when the cut-off value is set at p<1%, specificity increased to 81%, since 13 out 16 patients who did not fulfill DIS MRI criteria did not present any red-colored quadrant either. These findings reflect that OCT seems to have high sensitivity and moderate specificity for predicting DIS MRI. The sensitivity of DIS MRI criteria was higher than the criteria based on the finding of OCB in CSF plus two MRI lesions for DIS. However, this alternative criterion based on OCB detection yielded a slightly higher specificity than DIS-MRI at p<5% cut off value. These data therefore reinforce the role of CSF study in MS diagnosis.

In our series, 57% of CIS patients presented positive OCB. This result is in line with the proportions of CIS patients with positive OCB reported in previous studies. Tintore et al. [11] found that 61% of patients had positive OCB, considering that 41% of those with normal MRI did not undergo a lumbar puncture. Masjuan et al. [7] reported that 63% of patients were positive for OCB (7 out of 52 presented a normal scan and 7 had negative OCB) and Rojas et al. [42] detected positive OCB in 53% of CIS patients.

The present study is limited to a relatively small sample size. A larger sample was not achieved probably due to the fact that patients were included in the study within the first 4 weeks after the first event, and additionally, CSF and OCT had to be available before the initiation of treatment with corticosteroids in order to avoid false negative results owing to the effect of treatment. However, patients usually receive treatment before OCT and CSF analyses are performed when they arrive at the emergency room and the time elapsed until both examinations are performed is generally 6 weeks in routine clinical practice. In our study, patients underwent OCT, MRI and CSF examination within the two first months after the first event.

In the view of evidence, the relationship between RNFL thinning in CIS and progression to MS is still unclear, since only long-term follow-up will determine if these changes are clinically relevant. Therefore, prospective studies with long-term follow-up would be needed to establish the prognostic value of baseline OCT findings in patients with CIS, when dissemination in time and CDMS diagnosis could be determined.

In conclusion, axonal damage measured by OCT is present in any type of CIS, even in myelitis forms, not only in ON as seen up now. Although OCT has shown to detect axonal damage in very early stages of disease, its capacity to predict conversion in CDMS has not yet been demonstrated. Additional longitudinal studies would establish its role as prognostic marker of neurological disability. Thus, OCT could represent a potential tool that could be used to detect and monitor axonal protective effects of new neuroprotective therapies.
